# Spatiotemporal shifts in floristic composition under afforestation and climate variability in the sacred sites of Makkah, Saudi Arabia

**DOI:** 10.3897/BDJ.14.e186353

**Published:** 2026-04-07

**Authors:** Mohamed Fadl, Walaa Hassan, Shereen Magdy Korany, Emad Alsherif

**Affiliations:** 1 Department of Botany and Microbiology, Faculty of Science, Beni-Suef University, Beni-Suef, Egypt Department of Botany and Microbiology, Faculty of Science, Beni-Suef University Beni-Suef Egypt; 2 Department of Biology, College of Science, Taif University, Taif 21944, Saudi Arabia Department of Biology, College of Science, Taif University Taif 21944 Saudi Arabia; 3 Department of Biology, College of Science, Princess Nourah bint Abdulrahman University, P.O. Box 84428, Riyadh 11671, Saudi Arabia Department of Biology, College of Science, Princess Nourah bint Abdulrahman University P.O. Box 84428, Riyadh 11671 Saudi Arabia

**Keywords:** floristic composition, desert vegetation, afforestation impact, climate change, invasive species, species turnover

## Abstract

This study aimed to detect shifts in the floristic composition of the holy sites of Makkah over the past three decades, with special emphasis on the impact of afforestation and climate change. Results from a study carried out thirty years ago were compared with a contemporary survey (excluding afforested habitats) to isolate the impacts of climate change. An afforested area was compared to a nearby natural habitat to evaluate the effects of afforestation. The results show that the current floristic composition includes 116 species from 81 genera and 36 families. Afforestation significantly changed species composition (J = 0.19), mostly by replacing native desert taxa with invasive and disturbance-tolerant species. In addition, the proportional representation of life forms and chorotypes shifted substantially. Over the previous three decades, there has been a change in the amount of rainfall and the monthly average temperatures. While species richness increased by 90.9% compared to the 1989–1991 survey, (excluding afforested habitats), with high species turnover (82%, calculated as 1 – Jaccard similarity = 0.82) and 66% of the original taxa no longer recorded. Overall, the data show that both afforestation and climate change have significantly altered the floristic structure of the study area, emphasising the importance of management techniques targeted at limiting the establishment and spread of invasive species.

## Introduction

To sustain life on Earth and preserve ecological balance, flora is crucial; as the basis of the food chain. Fundamental alterations in ecosystem structure and function brought about by afforestation include modifications to productivity, microclimate, shading, nitrogen cycling and water balance, all of which have the potential to affect floristic composition. Floristic composition may be altered by human activities, such as afforestation (Khan et al. 2022). As planted trees provide safe microhabitats that are more favourable for seed germination and/or seedling recruitment than their immediate surroundings, supplemental irrigation helps other plant species grow and develop beneath their canopy (Bruno et al. 2003).

Global biodiversity has changed dramatically due to climate change, which is increasingly worrying governments and environmentalists alike (Ma et al. 2025). Most terrestrial ecosystems are dominated by higher plants, whose diversity and distribution strongly influence the range and diversity of many other organisms (Avolio et al. 2019). Many previous studies documented that plant species are often sensitive to a wide range of environmental changes, frequently affecting local species pools or plant communities (Hedwall and Brunet 2016, Sun et al. 2022). Due to cultural and religious safeguards that limit resource extraction and disruption, sacred sites, including holy places in Islamic contexts, often act as de facto refugia for biodiversity (Khan et al. 2022). Muslim graveyard groves in northwest Pakistan support species conservation through customary veneration while maintaining high plant diversity and offering a variety of ecosystem services, medical, nutritional and spiritual (Khan et al. 2022). In the same vein, Makkah's sacred places present a rare chance to study long-term floristic changes under the combined effects of climate variability and specific human interventions like afforestation. Thus, a primary objective should be to closely monitor any variations in vascular plant diversity and frequency (Finderup Nielsen et al. 2019). The Kingdom of Saudi Arabia is amongst the countries most vulnerable to climate change because of its continental climate, which features cold winters, extremely hot summers and irregular rainfall (Al-Wabel et al. 2020). Nevertheless, a lot of research has been done on the Mediterranean and its neighbouring regions in recent years (Lee et al. 2017). The literature and scientific understanding of climate and climate change in Saudi Arabia remain insufficient, scattered and fragmented (Alkolibi 2002, Alsherif and Almaghrabi 2022).

In the last three decades, the distribution and number of species have undergone major changes and at least one species-level extinction has been associated with climate change. Recently, many scientists have focused on predicting and modelling the effects of climate change on different areas (Zhang et al. 2025). Therefore, monitoring changes in the variety and abundance of vascular plants should be a key priority. During the Hajj season, the air temperature in the current study area may reach 50°C; hence, to protect visiting pilgrims from the risks of direct sun exposure, irrigation is carried out using motor-driven pumps to obtain groundwater supplies. Therefore, maintaining the biodiversity of these arid regions is essential to meeting international conservation goals, such as those set forth by the Convention on Biological Diversity. The current study offers the first long-term floristic assessment, disentangling the effects of climate change in the sacred sites of Makkah, based on a 30-year temporal comparison and functional (life-form and chorotype) analyses, as well as evaluating the effect of afforestation.

## Material and methods

**Study area**

**Figure 1.**
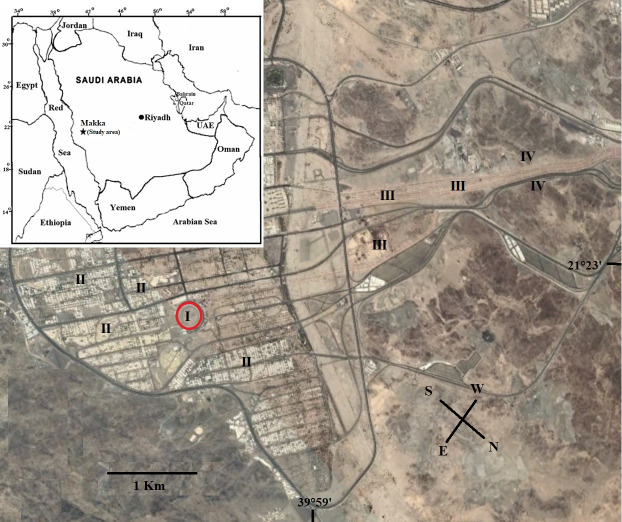
Geographic location and site map of the study area in Makkah, Saudi Arabia. The inset map (top left) indicates the study site's position within the Arabian Peninsula. The four sampled habitats: (I) Al-Rahmah Mountain, (II) Arafat Plain (afforested habitat), (III) Muzdalifa Plain (non-afforested habitat) and (IV) Muzdalifa Slope (granite rocky mountainous slope).

Makkah is a Saudi Arabian city located in the Hijaz Region, about 80 kilometres from Jeddah (Fig. 1). It is located between 21° 26' N and 39° 46' E and its elevation is between 240 and 277 m (a.s.l.) and surrounded by nearly continuous granite and granite gneiss ridges, which are primarily Precambrian crystalline rocks. The plant cover in the research region differs significantly between different sites due to the differences in terrain and soil characteristics, which undoubtedly affect how easily the various habitats can obtain water. The ground is deep, comprised, in some places, of varied layers of alluvium with various textures (Fayed and Zayed 1989). The alluvial deposits range in thickness from a thin mantle to valleys that have been tilled down to a depth of several metres. In other places, there are large boulders and gravel covering the ground. Since most of the soil is shallow and mixed with gravel, only a thin layer of vegetation can grow on it. As in most arid areas, rainfall is identified by rarity, irregularity and variability in both space and time. High air temperatures are common, especially in the summer. The typical monthly temperature for Makka ranges from 23.2°C in January to 36.3°C in July. The average high and low temperatures are 47.5°C and 25.6°C in June and 33.8°C and 15.6°C in January, respectively. Table 1 shows some climatic data about the study area during the two studies. The Jeddah station contains the longest continuous, quality-controlled meteorological dataset available for the region and has been extensively used in prior ecological studies of western Saudi Arabia. We recognise this as a potential constraint.

**Samples collection and identification**

**Figure 2.**
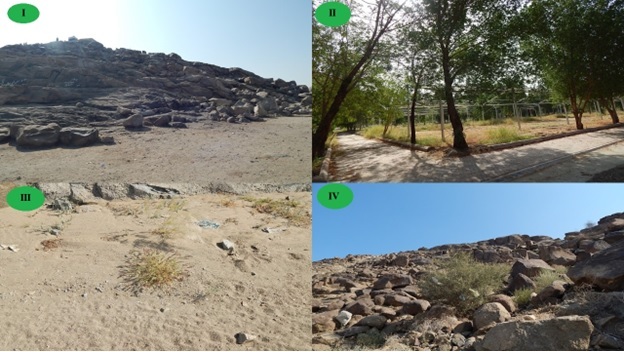
Representative views of the main habitats surveyed in the study area: (I) Al-Rahma Mountain (granite rocky outcrop); (II) Arafat Plain (afforested habitat); (III) Muzdalifa Plain (non-afforested habitat); and (IV) Muzdalifa Slope (granite rocky slope).

The studied area was divided into four different habitats. I: Al-Rahmah Mountain, the most notable location, is Arafat (or Arafa), which is distinguished by Al-Rahma Mountain, a small granite rocky mountain with a 50-metre elevation. II: Arafat Plain (afforested habitat), III: Muzdalifa Plain (non-afforested habitat) and IV: Muzdalifa Slope, a granite rocky mountainous slope (Fig. 2). Plant samples were gathered from 10 stands: one stand for Al-Rahmah Mountain, four stands for Arafat Plain (afforested habitat), three stands for Muzdalifa Plain (non-afforested habitat) and two stands for Muzdalifa Slope. Floristic sampling was carried out by utilising a stratified stand-based strategy to capture the spatial and environmental variation of Makkah's hallowed locations. The sampling stands were chosen to represent the principal land-use types (natural floristic composition vs. afforested areas), topographic settings and disturbance gradients in the study area. Each stand was positioned to maintain relative homogeneity in soil type, slope and vegetation structure, spanning roughly 100 × 100 m. Stands were arranged amongst the study sites in a purposeful-random manner, with a minimum distance between stands to ensure spatial independence. Each stand was surveyed 3–5 times between March 2021 and April 2022, timed to capture peak growth after major rainfall events and to include both early- and late-season species. Effort was standardised across habitats by allocating similar total survey time per stand (2 person- one hour per stand across visits). The historical survey (Abdelghani 1993) followed a comparable multi-season approach over two years, though exact visit numbers per stand are not detailed in the source; we assume comparable effort, based on the reported methodology. To maximise detectability and reduce false negatives, particularly for ephemeral annuals, surveys were conducted multiple times per stand during the growing season, following best practices for arid/semi-arid floristic inventories. Presence-absence data were used to focus on occurrence rather than abundance, which is less sensitive to observing differences or seasonal variation in cover. This strategy allowed for a robust assessment of changes in species composition while reducing the confounding effects of microhabitat variability. To ensure comparability between the historical (Abdelghani 1993) and current datasets, species names were cross-checked and harmonised using the following steps: (1) verification against updated regional floras (Collentette (1999) and Chaudhary 2001); then they were placed in the Biology Department's Herbarium at Taif University (TUH). The placement of the regenerative buds during the adverse season and parts that were shed were utilised to detect the life forms of the recognised species (Raunkiaer 1934). According to Zohary (1973)and Wickens (1976), the biogeographic affinities of the investigated species were ascertained at every elevation belt. The rules and standards that the Wildlife Service and the Department of Environmental Affairs when collecting plant samples were followed.

**Assessing how afforestation and climate change affect floristic composition**

To determine the impact of afforestation on the floristic composition, we compared the Arafat Plain (afforested habitat) with the Muzdalifa Plain (non-afforested habitat). This was done as part of the current survey because only the Arafat Plain was cultivated. We compared the recorded flora of a previous study, which was conducted from March 1989 to April 1991 (El-Ghani 1993), with the second study conducted by the current authors from March 2021 to April 2022 after excluding the afforested habitat where the trees were planted to identify the impact of climate change on the floristic composition.

**Statistical analysis**

Hierarchical agglomerative clustering was performed on the presence-absence (incidence) matrix of species across habitats and surveys, including the historical dataset, to visualise floristic relationships by using Statistica statistical software programme version 8 (Ward 1963). To ensure compatibility with binary data and Ward's minimum variance criterion, we calculated Jaccard dissimilarity. It is recommended for presence-absence floristic data, as it excludes double absences and focuses on shared presences while avoiding distortions from the double-zero problem inherent in Euclidean distance on incidence matrices. The formula defines this index:

\begin{varwidth}{50in}\begin{equation*}
            J(A,B)=(∣A∩B∣)/(∣A∪B∣)
        \end{equation*}\end{varwidth} ,

where A and B represent the sets of species recorded at two different time periods (or land-use conditions), \begin{varwidth}{50in}\begin{equation*}
            A∩B
        \end{equation*}\end{varwidth} is the number of species common to both assemblages and \begin{varwidth}{50in}\begin{equation*}
            A∪B
        \end{equation*}\end{varwidth} is the total number of species observed across both assemblages. The index ranges from 0 (no shared species) to 1 (identical species composition). Jaccard's index is especially useful for presence/absence data and historical comparisons when abundance data are lacking.

## Results

**Taxa composition and their distribution**

The results of recent and old studies showed that the holy sites included 116 species from 81 genera and 36 families (Appendix). Only four families, Amaranthaceae, Zygophyllaceae, Compositae and Poaceae, comprised 37.6% of all the species identified in the two studies. Poaceae was the largest family, with 13 genera and 19 species, followed by Zygophyllaceae, with three genera and eight species, while Papilionaceae, Amaranthaceae and Asteraceae were represented by six species each. With five species, *Cleome* and *Zygophyllum* were the largest genera, followed by *Cenchrus*, *Euphorbia* and *Pulicaria* with four species each, while 76.8% of the recorded genera were represented by only one species (Table 2). *Aerva javanica*, *Senna italica*, *Dipterygium glaucum*, *Forsskaolea tenacissima* and *Tetraena simplex* are five omnipresent species that were recorded in all habitats in the recent survey as well as in the old study. *Calotropis procera*, *Euphorbia granulata*, *Abutilon pannosum*, *Boerhavia diffusa*, *Aristida mutabilis*, *Cenchrus ciliaris*, *Cenchrus pennisetiformis*, *Panicum turgidum* and *Stipagrostis plumosa* were found in over 80% of the habitats under study (see Suppl. material 1) and more than 42% of the species were found in only one habitat. The Muzdalifa Slope had the highest species, genera and families’ counts, followed by Arafat Plain (afforested habitat), while Alrahma Mountain showed the lowest species numbers. Some species were restricted to specific habitats. Alrahma Mountain was the only location where nine species were found: *Heliotropium arbainense*, *Launaea intybacea*, *Cuscuta hyalina*, *Andrachne aspera*, *Cenchrus echinatus*, Pennisetum glaucum, *Ochradenus baccatus*, *Hyoscyamus albus* and *Fagonia bruguieri*, while thirty-two species were recorded only on Muzdalifa Slope, the most common of which were *Tamarix aphylla*, *Euphorbia cuneata*, *Hibiscus deflersii*, *Maerua crassifolia*, *Vachellia tortilis* and *Capparis decidua*.

**Life forms and their distribution**

**Figure 3.**
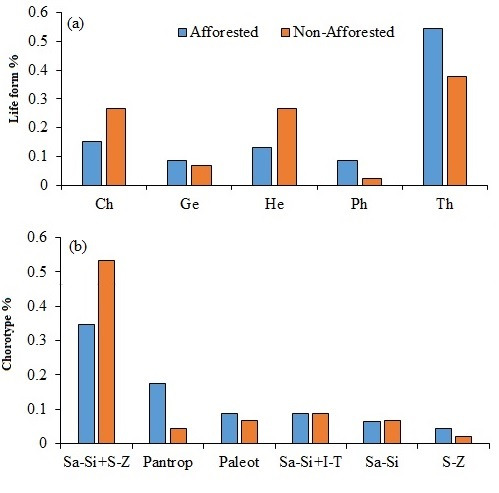
Proportional representation of plant life forms across the different habitats recorded in the recent survey. Afforested = Arafat Plain; Al-Rahma M = Al-Rahma Mountain; Non-afforested = Muzdalifa Plain; M slope = Muzdalifa Slope. Life-form categories follow Raunkiaer’s classification: Ch = chamaephytes, Ge = geophytes, He = hemicryptophytes, Ph = phanerophytes, Th = therophytes.

Five life forms were recorded in old and recent surveys (Fig. 3). The most represented life form was therophytes (41.1%), followed by chamaephytes (25.7%), hemicryptophytes (13.7%) and phanerophytes (11.9%), while geophytes exhibited the lowest life form (7.6%). Regarding life form distribution in the different habitats, the highest proportions of therophytes and geophytes were recorded in the afforested habitat, which were 54.3% and 8.6%, respectively. Fig. 3 shows that the lowest proportion of therophytes was recorded on Muzdalifa Slope (34.9%).

**Chorological affinities and their distribution**

The documented chronological elements of the area under study are shown in Table 2. Over 60.6% of the species that were recorded were biregional or pluriregional, with the Saharo-Sindian species accounting for the majority (44.4%), while 3.4% of all species were cosmopolitan. Following the Neotropical components, which each accounted for 9.4% of the monoregional elements, the Saharo-Sindian species accounted for almost 11%. Furthermore, the Saharo-Sindian components had the greatest bioregional species. Sa-Si/S-Z, Sa-Si and cosmopolitan elements recorded their highest numbers in the non-afforested habitat, according to the distribution of chorotypes (Table 2). At the same time, their numbers were larger in the recent research than in the previous one.

**Effect of afforestation on floristic composition**

**Figure 4.**
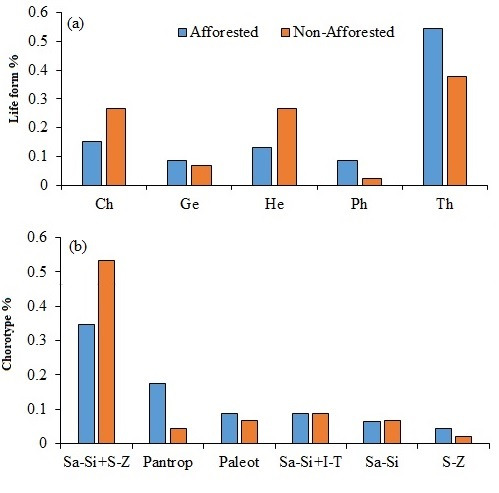
Life forms (a) and chorotype (b) of the recorded species in the afforested and non- afforested habitats (recent survey).

We compared the recent floristic compositions of Rahma Arafat (afforested habitat) with Muzdalifa Plain (non-afforested habitat) to demonstrate how afforestation affects floristic compositions in the recent study. The findings revealed a considerable difference in species composition, but no discernible difference in species number. More than 64% of the species found in the afforested habitat were absent from the non-afforested habitat; in contrast, 61.3% of the species found in the non-afforested habitat were absent from the afforested habitat. *Trianthema portulacastrum*, *Prosopis juliflora*, Amaranthus blitum ssp. Oleraceus, *Euphorbia prostrata*, *Portulaca oleracea*, *Cynodon dactylon*, *Chloris Gayana*, Phyllanthus tenellus var. arabicus and *Cyperus longus* were the most prevalent species in the Arafat Plains that were absent in the non-afforested habitat. On the other hand, *Senegalia hamulosa*, *Euphorbia arabica*, *Cleome paradoxa*, *Indigofera spinosa*, *Tephrosia nubica*, *Panicum turgidum* and *Tribulus terrestris* were the species most prevalent in non-afforested habitat and non-existent in the afforested habitat. Four species, *Aerva javanica*, *Calotropis procera*, *Senna italica* and *Dipterygium glaucum*, were the most prevalent species, accounting for around 18.4% of all species found in both habitats (Appendix). In addition, the proportion of therophytes was higher in the non-afforested habitat than in the afforested habitat; in contrast, the proportions of hemicryptophytes and chamaephytes were higher in the non-afforested habitat than in the afforested habitat (Fig. 4). Therophytes were higher in the Arafat Plain (afforested habitat) than in the Muzdalifa Plain (non-afforested habitat). Palaeotropical, pantropical and Sudano-Zambesian elements exhibited higher proportions in the Arafat Plain (afforested habitat) than those in the Muzdalifa Plain (non-afforested habitat). In contrast, the biregional Saharo-Sindian/Sudano-Zambesian showed higher proportions in non-afforested habitat than those in afforested habitat by 38.6%.

**Effect of climate change on floristic composition**

**Figure 5.**
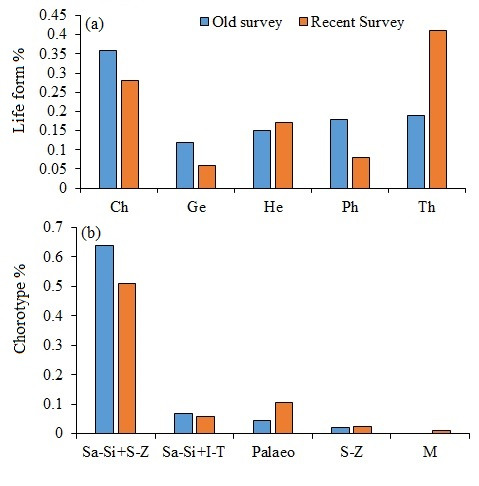
Life forms (a) and chorotype (b) of the recorded species in the old survey and recent survey.

**Figure 6.**
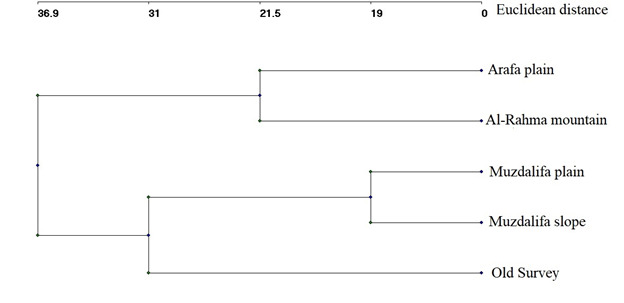
Hierarchical clustering of the studied habitats, based on floristic composition using presence–absence (incidence) data. The dendrogram was generated using Ward’s minimum variance method with Euclidean distance as the measure of linkage distance.

Except for the Arafat Plain (because of afforestation), we compared the old and new studies to demonstrate how climate change affects floristic compositions. The number of species found in the current study was greater than those found in the previous study by 90.9%. While 34% of the species from the previous study were not present in the current one, at the same time, over 72% of the species from the recent study were not recorded in the previous one (Table 2). Trees, *Capparis decidua*, *Maerua crassifolia*, *Ziziphus spina-christi* and *Tamarix aphylla* make up 26.6% of the species that were identified in the prior survey, but were not included in the recent survey. In contrast, the most prominent species that was recorded in the present study was the invasive *Prosopis juliflora*, which was recorded in both Arafat Plain and Alrahma Mountain. *Commiphora gileadensis*, *Conocarpus lancifolius*, *Euphorbia cuneata*, *Hibiscus deflersii* and *Melica persica* were the most frequently recorded species in the recent survey and absent from the oldest one (Suppl. material 1). The proportion of the therophyte in the recent survey was two-fold that of the old survey and hemicryptophytes recorded an 80% increase in the recent survey compared to those of the old survey. In contrast, chamaephytes, geophytes and phanerophytes were more than those recorded in the recent survey by 28.5%, 100% and 125%, respectively (Fig. 5). When comparing the proportion of the chorological elements of the old study with the recent one, the Palaeotropical, Sudano-Zambesian and Mediterranean elements of the recent study were higher than those recorded in the old one. In contrast, the Biregional Saharo-Sindian/Sudano-Zambesian and Saharo-Sindian/Irano-Turanian of the recent survey showed lower proportions than those recorded in the old one. A decrease in phanerophytes was recorded in the recent study by more than 50%. The Ward classification generated a dendrogram that divided the various habitats, including the old survey, into two main categories (Fig. 6). The first group consisted of the old survey, Muzdalifa Plain (non-afforested habitats) and Muzdalifa Slope, which was divided into two subgroups: one included the old survey and the other included Muzdalifa Plain (non-afforested habitats) and Muzdalifa Slope, which was divided into two subgroups, each representing one habitat. The second principal category consisted of the Arafat Plain (afforested habitat) and Al-Rahma Mountain. Jaccard's similarities (Table 3) amongst different habitats, including the old survey, revealed that the highest similarity index was recorded between the Muzdalifa Plain (non-afforested habitat) and the Muzdalifa Slope; in contrast, the lowest similarity index was recorded between the old survey and the Arafat Plain (Table 3). The Jaccard Similarity Index between the current survey (excluding afforested habitats) and the historical survey was 0.18, corresponding to a species turnover of 82% (1 – J). Over the course of the three decades, most of the original floristic composition has either been lost or replaced, as seen by this low value, which shows a high degree of species turnover (82%); turnover values refer to comparisons excluding afforested stands to isolate climate effects. The afforested Arafat Plain and the wild Muzdalifa Plain, on the other hand, had a similarity of 0.19, suggesting that human activity has produced a unique biological community that shares fewer than 20% of its species with nearby natural environments.

## Discussion

**Floristic Diversity and Taxonomic Patterns**

The predominance of Poaceae, Zygophyllaceae and Amaranthaceae is consistent with the Arabian Peninsula's broader phytogeographical patterns (Al-Nafie 2008, Collentette 1999). High ecological tolerance and effective anemochorous (wind-dispersed) seed dispersal are key factors contributing to the persistence of Poaceae. Notably, the highest species richness was recorded on the Muzdalifa Slope. This pattern can be explained by the “rock-mulch” effect in arid environments, granite outcrops acting as water harvesters. Runoff from smooth rock faces concentrates moisture within rock crevices, forming stable micro-refugia for plants (Shakdofa et al. 2024).

**Impact of Afforestation**

Our findings show that the local plant community has undergone fundamental restructuring due to afforestation. The low Jaccard Similarity Index (J = 0.19) between afforested and natural habitats indicates that the native community has been replaced rather than simply supplemented by human intervention. Although broad-leaved trees offer cooling and shade (Cerabolini et al. 2009), the irrigation and soil disturbance they have made it easier for exotics such as *Prosopis juliflora* to invade. The appearance of halophytic species like *Suaeda aegyptiaca* in afforested regions points to possible limited secondary salinisation, which is probably caused by high evaporation rates typical of Makkah's hyper-arid climate in conjunction with ongoing supplemental irrigation.

The shift towards salt-tolerant taxa is consistent with patterns seen in other afforested arid environments where irrigation encourages halophyte dominance over native xerophytes, even though direct soil salinity measurements, such as electrical conductivity or ion concentrations, were not carried out in this study. This conclusion draws attention to a potential unintended effect of present afforestation practices, necessitating more soil monitoring to verify salinity gradients and guide modified management (Richardson and Rejmánek 2011, Rundel et al. 2014). Furthermore, afforestation typically involves soil disturbance, road construction and repeated human activity, all of which enhance propagule pressure and create invasion pathways. Once established, invasive trees and shrubs, such as *Prosopis juliflora* in arid regions, can exacerbate invasion dynamics by altering fire regimes, suppressing native regeneration via allelopathy and monopolising water resources, resulting in long-term shifts in community structure and ecosystem functioning (van Wilgen et al. 2025). As a result, afforestation may increase the diversity of local plant species while also promoting functional simplicity and the loss of native specialists, emphasising the importance of distinguishing between tree cover growth and actual ecological restoration.

**Climate Change and Species Turnover**

The 90.9% increase in species richness and the 60% loss rate are two startling results of this study. Despite the short 34-year geological timeframe, 66% of the species originally identified are no longer present in the current floristic record. The rise in richness is probably a "pulse" reaction to the current survey period's somewhat lower average temperatures and more rainfall (Table 1). Therophytes or annuals are found in the seed bank in arid settings and only appear during suitable windows. On the other hand, the elimination of chamaephytes and perennial phanerophytes (a decrease of 28.5% and 125%, respectively) is a concerning indication of long-term climate stress. The loss of long-lived perennials, which act as the "scaffolding" of the ecosystem, suggests that "tipping points" in temperature and drought frequency are endangering the fundamental structure of the desert community, even when annuals offer a brief greening impact (Berdugo et al. 2022). Increases in α-diversity may disguise reductions in other aspects of biodiversity, such as evenness and β-diversity, which are essential for ecosystem resilience and niche complementarity (Soininen et al. 2011); a community dominated by a few widely-spread weeds or generalists may have a high species diversity, but limited functional redundancy, tolerance to new stresses and impaired ecosystem services. This phenomenon has been observed in long-term studies where local species richness rose, while regional specificity and specialist species occupancy decreased, indicating that richness alone is an inadequate metric for ecosystem health in a setting of global change and anthropogenic disturbances (Finderup Nielsen et al. 2019). Attribution to climate change alone requires caution. Over the 30+ year interval, other factors, including increased anthropogenic disturbance (e.g. pilgrim-related trampling and infrastructure development around sacred sites), altered management practices, propagule pressure from invasives like *Prosopis juliflora* and potential hydrological changes, may have contributed to or confounded these patterns. Long-term studies in arid regions frequently show that such drivers interact with climate, often amplifying turnover or favouring generalist species. Our stratified sampling and exclusion of afforested habitats aimed to minimise land-use confounding for the temporal comparison, yet residual effects from non-climatic drivers cannot be fully ruled out without additional controls or process-based data. Future research incorporating repeated soil/microclimate monitoring and disturbance histories would strengthen causal inference.

## Conclusions

Over the past thirty years, Makkah's sacred places have experienced a significant change in their floral landscape. This study demonstrates that the ecological integrity of the area is in danger even if nominal species richness has increased by 90.9%, most likely due to inter-annual climate changes and higher rainfall throughout the study period. With 66% of the original native taxa no longer found in natural environments, the incredibly low Jaccard Similarity Index (J = 0.18) shows a significant shift in species composition. There are two sides to afforestation. Although it gives pilgrims the necessary thermal comfort, it has unintentionally acted as a bridgehead for invasive species, like *Trianthema portulacastrum* and *Prosopis juliflora*. Additionally, the development of halophytic communities is proof that the irrigation of farmed regions has caused localised secondary salinisation. The predominance of transitory therophytes and the decrease in native phanerophytes and chamaephytes indicate that the vegetation in the area is becoming more uniform and fleeting. The botanical legacy of these revered granite outcrops is being drastically altered by both human activity and climate change.

## Recommendations

The study recommends the use of indigenous, drought-tolerant species, such as *Vachellia tortilis*, *Ziziphus spina-christi* (native variants) and *Maerua crassifolia*, should be mandated. These species provide shade while supporting native insect and bird populations. To stop *Prosopis juliflora* from spreading from the Arafat lowlands into the nearby mountain slopes, where it poses a threat to local endemics, immediate biological or mechanical control operations are required.

## Supplementary Material

EA82539F-80EF-563C-AB00-8BC324BEF084 10.3897/BDJ.14.e186353.suppl1 Supplementary material 1 Appendix: Presence/absence of plant species recorded during the old and recent surveys, with life-form categories and chorological affinities Data type Occurrence Brief description The full dataset, including presence/absence records across different survey periods, life forms and chorological affinities, has been deposited in the Zenodo repository to ensure open access and long-term preservation. The persistent link is available at: DOI 10.5281/zenodo.18989107 File: oo_1513873.docx https://binary.pensoft.net/file/1513873 Mohamed A. Fadl, Shereen Magdy Korany, Walaa A. Hassan, Emad A. Alsherif

## Figures and Tables

**Table 1.** Temperature, precipitation and humidity of the study area during recent and old (in parenthesis) surveys. The data were obtained from the meteorological station in Jeddah.

	January	February	March	April	May	June	July	August	September	October	November	December
Mean temperature (°C)	21.3 (25.5)	23.3 (23.6)	25.6 (28.7)	28.9 (30.6)	32.2 (34.1)	33.6 (35.6)	33.6 (34.9)	33.3 (35.3)	32.5 (34.4)	29.6 (31.5)	25.8 (27.8)	23 (25)
Min. Temperature (°C)	15.8 (10.2)	17.3 (17.6)	19 (22.2)	22.2 (24.2)	24.8 (27.2)	26 (27.2)	26.8 (27.5)	26.9 (28.8)	26 (28.2)	23.4 (24.7)	20.1 (22.6)	17.3 (19.7)
Max. temperature (°C)	27 (31.7)	29.8 (30.7)	32.5 (36)	35.7 (37.7)	39.2 (41.4)	40.8 (43.9)	40 (41.8)	39.5 (42)	39 (41.7)	36.2 (39.4)	31.6 (33.9)	28.6 (30.7)
Precipitation (mm)	43 (3)	4 (0)	10 (0)	12 (2)	6 (0)	0 (0)	0 (0)	1 (0)	3 (8)	6 (0.01)	20 (23.5)	25 (69)
Humidity (%)	54 (63)	47 (54)	39 (49)	34 (45)	27 (41)	24 (36)	27 (32)	36 (37)	39 (45)	44 (51)	51 (62)	54 (57)

**Table 2.** Number of taxa, life forms and chorotype in old and recent surveys. The life forms are Ph, phanerophytes; Ch, chamaephytes; G, geophytes; He, hemi-cryptophytes and Th, therophytes. The chorotypes are: COSM, cosmopolitan AM, American; IT, Irano-Turanian; ME, Mediterranean; SA, Saharo-Arabian; SU, Sudano Zambezian and TR, Tropical.

	Recent survey				Recent survey	Old survey	Both surveys
	Afforested	Al-Rhama Mountain	Muzdalifa Slope	Non-afforested			
Taxa distribution							
Number of species	46	38	45	63	104	43	116
Number of genera	38	34	32	51	71	40	82
Number of families	22	19	21	27	35	22	36
Life form distribution							
Therophytes	25	19	17	22	46	8	48
Chamaephytes	7	8	12	19	27	16	30
Hemicryptophyte	6	6	12	12	16	6	16
Phanerophyte	4	2	1	5	9	8	14
Geophytes	4	3	3	5	8	5	9
Chorotype distribution							
Sa-Si	3	2	3	9	12	3	14
Neotrop	8	6	2	2	10	1	11
Palaeo	4	4	3	4	9	2	9
S-Z	2	1	1	0	2	1	2
M	1	0	0	0	1	0	1
Sa-Si+S-Z	16	15	25	30	43	28	52
Sa-Si+I-T	4	3	4	2	5	3	7
Sa-Si+M	1	1	1	1	2	1	2
M+I-T	1	0	0	1	2	1	2
Palaeo+Neotrop	0	0	0	1	1	0	1
Sa-Si+I-T+S-Z	4	2	3	5	6	2	6
Sa-Si+M+I-T	0	1	0	1	2	0	2
Sa-Si+M+I-T+S-Z	1	1	1	1	2	1	2
Palaeo+M+I-T	0	0	1	1	1	0	1
Sa-Si+M+ S-Z	0	0	1	1	1	0	1
Cos	1	2	0	4	4	0	4

**Table 3.** Jaccard similarities between different habitats based on their floristic compositions.

	Arafa Plain	Al-Rhama Mountain	Muzdalifa Plain	Muzdalifa Slope	Old surveys
Arafa Plain	1				
Al-Rhama Mountain	0.3125	1			
Muzdalifa Plain	0.2133	0.2576	1		
Muzdalifa Slope	0.2111	0.2949	0.4795	1	
Old survey	0.1688	0.2059	0.2361	0.2892	1
