# Supplementary material for: Spatiotemporal shifts in floristic composition under afforestation and climate variability in the sacred sites of Makkah, Saudi Arabia
Source: Biodivers Data J. 2026 Apr 7;14:e186353. doi: 10.3897/BDJ.14.e186353 (PMC13080384; doi:10.3897/BDJ.14.e186353)
Supplement: Supplementary material 1 — Appendix: Presence/absence of plant species recorded during the old and recent surveys, with life-form categories and chorological affinities [file bdj-14-e186353-s001.docx]

**Appendix**. Presence/absence of plant species recorded during the old and recent surveys, with life-form categories and chorological affinities. The life forms are Ph, phanerophytes; Ch, chamaephytes; G, geophytes; He, hemi-cryptophytes and Th, therophytes. The chorotypes are: COSM, cosmopolitan AM, American; IT, Irano-Turanian; ME, Mediterranean; SA, Saharo-Arabian; SU, Sudano Zambezian and TR, Tropical

|  |  | **Old survey** | **Recent survey** | **Life form** | **Chorology** |
| --- | --- | --- | --- | --- | --- |
| **Acanthaceae** | *Blepharis ciliaris* (L.) B.L. Burtt. | 1 | 1 | Ch | Sa-Si+S-Z |
|  | *Dicliptera paniculata*(Forssk.) I.Darbysh. | 0 | 1 | Th | Palaeo |
| **Aizoaceae** | *Aizoon canariense* L*.* | 1 | 1 | Th | Sa-Si+S-Z |
|  | *Trianthema portulacastrum* L. | 0 | 1 | Th | Pantrop |
|  | *Trianthema triquetra*Willd. ex Spreng. | 0 | 1 | Th | Pantrop |
|  | *Zaleya pentandra* (L.) Jeffrey | 0 | 1 | He | Sa-Si+M+I-T+S-Z |
| **Amaranthaceae** | *Aerva javanica* Juss. | 1 | 1 | Ch | Sa-Si+S-Z |
|  | *Aerva lanata* (L.) Juss. | 0 | 1 | Ch | Sa-Si+S-Z |
|  | *Amaranthus blitum* subsp. *oleraceus* (L.) Costea | 0 | 1 | Th | Pantrop |
|  | *Amaranthus graecizans* L*.* | 0 | 1 | Th | Pantrop |
|  | *Amaranthus hybridus* L. | 0 | 1 | Th | Pantrop |
|  | *Chenopodium murale* L. | 0 | 1 | Th | Cos |
|  | *Suaeda aegyptiaca* (Hasselq,) Zoh*.* | 0 | 1 | Th | Sa-Si+ I-T |
| **Apiaceae** | *Apium graveolens* L. | 0 | 1 | He | Cos |
| **Apocynaceae** | *Calotropis procera* (Ait.) Ait.f. | 1 | 1 | Ch | Sa-Si+S-Z |
|  | *Leptadenia pyrotechnica* (Forssk.) Decne. | 1 | 1 | Ph | Sa-Si+S-Z |
|  | *Pergularia tomentosa* L. | 0 | 1 | Ch | Sa-Si+S-Z |
|  | *Rhazya stricta* Decne | 1 | 1 | Ch | Sa-Si+S-Z |
| **Astereaceae** | *Lactuca serriola* L. | 0 | 1 | Th | I-T+M |
|  | *Launaea intybacea* (Jacq.) Beauverd | 0 | 1 | Th | Sa-Si+S-Z |
|  | *Launaea massauensis*(Fres.) Chiov. | 0 | 1 | Th | Sa-Si+S-Z |
|  | *Pulicaria arabica* (L.) Cass | 0 | 1 | Th | Sa-Si+S-Z |
|  | *Pulicaria incisa* **subsp..** *Candolleana* E.Gamal-Eldin | 0 | 1 | Th | Sa-Si+S-Z |
|  | *Pulicaria inuloides* (Poir) DC. | 0 | 1 | He | Sa-Si |
|  | *Pulicaria undulata* (L.) C.A. Mey | 1 | 0 | Ch | Sa-Si+S-Z |
|  | *Sonchus oleraceus* L. | 0 | 1 | Th | Cos |
| **Boraginaceae** | *Heliotropium arbainense* Fresen. | 1 | 1 | Ch | Sa-Si+S-Z |
|  | *Heliotropium bacciferum* Forssk. | 1 | 1 | Ch | Sa-Si+S-Z |
|  | *Heliotropium ramosissimum* (Lehm.) DC. | 0 | 1 | Ch | Sa-Si+S-Z+ I-T |
|  | *Trichodesma africanum* (L.) Sm. | 1 | 1 | Ch | Sa-Si+S-Z |
| **Brassicaceae** | *Brassica tournefortii* Gouan. | 0 | 1 | Th | M +I-T + Sa-Si |
|  | *Farsetia longisiliqua* Decne. | 1 | 2 | Ch | Sa-Si+S-Z |
|  | *Morettia parviflora* Boiss. | 0 | 2 | Ch | Sa-Si+S-Z |
|  | *Schouwia purpurea* (Forssk.) Schweinf. | 0 | 1 | Th | Sa-Si |
| **Burseraceae** | *Commiphora gileadensis* C. Christ. | 0 | 1 | Ph | Sa-Si |
| **Caesalpinaceae** | *Senna holosericea* (Fresen) Greuter | 0 | 1 | He | Sa-Si+S-Z |
|  | *Senna italica* Miller | 1 | 1 | He | Sa-Si+S-Z |
| **Capparaceae** | *Dipterygium glaucum* Decne. | 1 | 1 | He | Sa-Si+ I-T |
|  | *Capparis decidua* (Forssk.) Edgew | 1 | 0 | Ph | Sa-Si+S-Z |
|  | *Maerua crassifolia* Forssk. | 1 | 0 | Ph | Sa-Si+S-Z |
| **Cleomaceae** | *Cleome austroarabica* D.F. Chamb.& Lam. | 0 | 1 | Th | Sa-Si |
|  | *Cleome chrysantha* Decne. | 0 | 1 | Ch | Sa-Si |
|  | *Cleome droserifolia* (Forssk.) Del. | 1 | 0 | Ch | Sa-Si |
|  | *Cleome paradoxa* R. Br. | 0 | 1 | Ch | Sa-Si+S-Z |
|  | *Cleome scaposa* DC. | 0 | 1 | Th | Sa-Si+S-Z |
| **Combretaceae** | *Conocarpus lancifolius* Engl. | 0 | 1 | Ph | Sa-Si |
| **Convolvulaceae** | *Cuscuta hyalina* Roth | 0 | 1 | Th | Sa-Si+S-Z |
|  | *Cuscuta campestris* Yuncker | 0 | 1 | Th | Pantrop |
| **Cucurbitaceae** | *Citrullus colocynthis* (L.) Schrader | 1 | 1 | He | Sa-Si+M |
|  | *Cucumis prophetarum* L. | 1 | 0 | He | Sa-Si+S-Z |
| **Cyperaceae** | *Cyperus longus* L. | 0 | 1 | Ge | M |
|  | *Euphorbia arabica*Hochst. & Steud. ex T.Anderson | 0 | 1 | He | Sa-Si+M+S-Z |
|  | *Euphorbia cuneata* Vahl | 0 | 1 | Ph | Sa-Si+S-Z |
|  | *Euphorbia granulata* Forssk*.* | 1 | 1 | Th | S-Z |
|  | *Euphorbia prostrata* Aiton | 0 | 1 | Th | Pantrop |
| **Lamiaceae** | *Ocimum forsskaolii* Benth. | 0 | 1 | Th | Sa-Si+S-Z |
| **Malavceae** | *Abutilon pannosum* (Forst.f.) Schlecht. | 1 | 1 | Ch | Sa-Si+S-Z |
|  | *Hibiscus deflersii* Schweinf. ex Cufod. | 0 | 1 | Ch | Paleot + Pant |
|  | *Malva parviflora* L. | 1 | 1 | Th | M+I-T |
|  | *Sida alba* L*.* | 0 | 1 | Ch | Sa-Si |
| **Meliaceae** | *Azadirachta indica* A. Juss. | 0 | 1 | Ph | Paleot |
| **Mimosaceae** | *Vachellia flava*(Forssk.) Kyal. & Boatwr. | 1 | 1 | Ph | Sa-Si+S-Z |
|  | *Senegalia hamulosa*(Benth.) Boatwr. | 0 | 1 | Ph | Sa-Si+S-Z |
|  | *Vachellia tortilis*(Forssk.) Galasso & Banfi | 1 | 1 | Ph | Sa-Si+S-Z |
|  | *Prosopis juliflora* (SW.) DC. | 0 | 1 | Ph | Pantrop |
| **Mollugonaceae** | *Mollugo cerviana* (L.) Seringe | 0 | 1 | Th | Palaeo +M+I-T |
| **Nyctaginaceae** | *Boerhavia diffusa* L. | 0 | 1 | He | Pantrop |
|  | *Boerhavia elegans* **subsp.** *elegans* Choisy | 0 | 1 | He | Sa-Si+S-Z |
|  | *Boerhavia sinuata* (Meikle) Greuter & Burdet. | 0 | 1 | He | Sa-Si+S-Z |
| **Papilionaceae** | *Astragalus vogelii* (Webb) Bornm. | 1 | 0 | Th | Sa-Si |
|  | *Crotalaria microphylla* Vahl. | 0 | 1 | Th | Sa-Si+S-Z |
|  | *Indigofera hochstetteri* Bak. | 0 | 1 | Th | Sa-Si+S-Z |
|  | *Indigofera spinosa* Forssk. | 1 | 1 | Ch | Sa-Si+S-Z |
|  | *Tephrosia nubica* (Boiss.) Bak. | 0 | 1 | Ch | Sa-Si+S-Z |
|  | Tephrosia purpurea (L.) Pers. | 0 | 1 | Th | Sa-Si+S-Z |
| **Phyllanthaceae** | *Andrachne aspera* Spreng. | 1 | 0 | Ch | Sa-Si+S-Z |
|  | *Phyllanthus tenellus* var. *arabicus* Müll.Arg | 0 | 1 | Th | Sa-Si+S-Z |
| **Plantaginaceae** | *Kickxia acerbiana* (Boiss.) Tackh.& Boulos | 0 | 1 |  | Sa-Si |
|  | *Lindenbergia indica* Vatke | 0 | 1 | Ch | Palaeo |
| **Poaceae** | *Aristida mutabilis* Trin. &Rupr. | 1 | 1 | Th | Palaeo |
|  | *Brachiaria leersioides* (Hochst.) Stapf | 0 | 1 | Th | Palaeo |
|  | *Cenchrus ciliaris* L. | 1 | 1 | He | Palaeo |
|  | *Cenchrus echinatus* L. | 0 | 1 | Th | Pantrop |
|  | *Cenchrus pennisetiformis*Steud*.* | 0 | 1 | Th | Sa-Si+I-T+S-Z |
|  | *Cenchrus setigerus* Vahl. | 0 | 1 | Ge | Sa-Si+S-Z |
|  | *Chloris gayana* Kunth | 0 | 1 | Th | Palaeo |
|  | *Cynodon dactylon* (L.) Pers. | 1 | 1 | Ge | Sa-Si+S-Z+I-T |
|  | *Dactyloctenium aegyptium* (L.) Willd. | 0 | 1 | Th | Palaeo |
|  | *Digitaria sanguinalis* (L.) Scop. | 0 | 1 | Th | Sa-Si+S-Z+I-T |
|  | *Eragrostis barrelieri* Dav. | 0 | 1 | Th | Sa-Si |
|  | *Panicum turgidum* Forssk. | 1 | 1 | Ge | Sa-Si+M +S-Z |
|  | *Pennisetum divisum* (Gmel.) Henr. | 1 | 0 | Ge | Sa-Si+S-Z |
|  | *Pennisetum glaucum* (L.) R.Br. | 0 | 1 | Th | Sa-Si |
|  | *Pennisetum macrourum* Trin. | 0 | 1 | Ge | S-Z |
|  | *Melica persica*Kunth | 0 | 1 |  | Sa-Si+M +I-T |
|  | *Stipagrostis ciliata* (Desf.) de Wint. | 0 | 1 | He | Sa-Si+S-Z |
|  | *Stipagrostis plumosa* (L.) Munro ex T. Anders. | 1 | 1 | Ge | Sa-Si+I-T+S-Z |
|  | *Tricholaena teneriffae*  (L.f.) Link. | 1 | 1 | Ge | Sa-Si+S-Z+I-T+M |
| **Polygalaceae** | *Polygala abyssinica* R.Br. ex Fresen. | 0 | 1 | Ch | Sa-Si+S-Z+I-T |
| **Portulacaceae** | *Portulaca oleracea* L. | 0 | 1 | Th | Cos |
| **Rhamnaceae** | *Ziziphus spina-christi* (L.) Willd | 1 | 0 | Ph | Sa-Si+S-Z |
| **Resedaceae** | *Ochradenus baccatus* Del. | 1 | 0 | Ph | Sa-Si+S-Z |
| **Scrophulariaceae** | *Anticharis arabica* Endl. | 0 | 1 | Th | Sa-Si |
| **Solanaceae** | *Hyoscyamus albus* L. | 0 | 1 | Th | Sa-Si + M |
|  | *Datura innoxia* Mill. | 1 | 0 | Th | Pant. |
|  | *Solanum incanum* L. | 1 | 0 | Ch | Sa-Si |
| ***Tamaricaceae*** | *Tamarix aphylla* (L.) Karst. | 1 | 0 | Ph | Sa-Si+S-Z |
| **Urticaceae** | *Forsskaolea tenacissima* L. | 1 | 1 | He | Sa-Si+S-Z |
| **Zygophyllaceae** | *Zygophyllum bruguieri*(DC.) Christenh. & Byng | 1 | 0 | Ch | Sa-Si+I-T |
|  | *Zygophyllum indicum*(Burm.f.) Christenh. & Byng | 1 | 0 | Ch | Sa-Si+I-T |
|  | *Zygophyllum olivieri*(DC.) Christenh. & Byng | 0 | 1 | Ch | Sa-Si+I-T |
|  | *Zygophyllum paulayanum*(J.Wagner & Vierh.) Christenh. & Byng | 0 | 1 | Ch | Sa-Si+I-T |
|  | *Tetraena* *simplex* (L.) Beier&Thulin | 1 | 1 | Th | Sa-Si+S-Z |
|  | *Tribulus macropterus* var*. macropterus* Boiss. | 0 | 1 | Th | Sa-Si+S-Z |
|  | *Tribulus pentandrus* Forssk. | 1 | 1 | Th | Sa-Si+S-Z |
|  | *Tribulus terrestris* L. | 0 | 1 | Th | Sa-Si+S-Z |
